# The potential role of infectious agents and pelvic inflammatory disease in ovarian carcinogenesis

**DOI:** 10.1186/s13027-017-0134-9

**Published:** 2017-05-18

**Authors:** Kasper Ingerslev, Estrid Hogdall, Tine Henrichsen Schnack, Wojciech Skovrider-Ruminski, Claus Hogdall, Jan Blaakaer

**Affiliations:** 1 Department of Gynaecology and Obstetrics, Odense University Hospital, Denmark, Soendre Blvd. 29, 5000 Odense C, Denmark; 2 Department of Pathology, Herlev and Gentofte Hospital, Denmark, Herlev Ringvej 75, 2730 Herlev, Denmark; 3 Gynaecologic Clinic, Copenhagen University Hospital, Denmark, Blegdamsvej 9, 2100 Copenhagen, Denmark; 4 Department of Pathology, Herlev Hospital, Denmark, Herlev Ringvej 75, 2730 Herlev, Denmark

**Keywords:** Ovarian cancer, Bacterial infection, Viral infection, Pelvic inflammatory disease, Carcinogenesis

## Abstract

**Background:**

The etiological cause of ovarian cancer is poorly understood. It has been theorized that bacterial or viral infection as well as pelvic inflammatory disease could play a role in ovarian carcinogenesis.

**Aim:**

To review the literature on studies examining the association between ovarian cancer and bacterial or viral infection or pelvic inflammatory disease.

**Methods:**

Database search through MEDLINE, applying the medical subject headings: “Ovarian neoplasms”, AND “Chlamydia infections”, “*Neisseria gonorrhoeae*”, “*Mycoplasma genitalium*”, “Papillomaviridae”, or “pelvic inflammatory disease”. Corresponding searches were performed in EMBASE, and Web of Science. The literature search identified 935 articles of which 40 were eligible for inclusion in this review.

**Results:**

Seven studies examined the association between bacterial infection and ovarian cancer. A single study found a significant association between chlamydial infection and ovarian cancer, while another study identified *Mycoplasma genitalium* in a large proportion of ovarian cancer cases. The remaining studies found no association. Human papillomavirus detection rates varied from 0 to 67% and were generally higher in the Asian studies than in studies from Western countries. Cytomegalovirus was the only other virus to be detected and was found in 50% of cases in a case-control study. The association between ovarian cancer and pelvic inflammatory disease was examined in seven epidemiological studies, two of which, reported a statistically significant association.

**Conclusions:**

Data indicate a potential association between pelvic inflammatory disease and ovarian cancer. An association between ovarian cancer and high-risk human papillomavirus genotypes may exist in Asia, whereas an association in Western countries seems unlikely due to the low reported prevalence. Potential carcinogenic bacteria were found, but results were inconsistent, and further research is warranted.

**Electronic supplementary material:**

The online version of this article (doi:10.1186/s13027-017-0134-9) contains supplementary material, which is available to authorized users.

## Background

Ovarian cancer (OC) is a major threat to female health, with a global prevalence of 239,000 cases in 2012 [1]. Due to the lack of symptoms in early stage OC, advanced disease is often present at the time of diagnosis [2].

Almost 90% of OC originates from epithelial cells, which have been thought to arise from the surface mesothelial lining of the ovaries. However, recent research has indicated that a proportion of serous epithelial OC could originate in precancerous lesions called “serous tubal intraepithelial carcinomas” (STICs) located in the fimbriated end of the fallopian tubes [3]. This is an important finding because previous theories of ovarian carcinogenesis have been unable to explain the fact that precancerous lesions have never been identified in the ovaries [3].

The female internal genitalia and the peritoneal cavity are accessible to outside pathogens through the genital tract. The mesothelial lining of the peritoneal cavity is identical in both sexes. However, primary serous peritoneal cancer, which may represent a continuum with serous OC [3], occurs almost exclusively in female patients [4]. This suggests that extrinsic factors could play a crucial role in ovarian carcinogenesis. Furthermore, epidemiological studies have demonstrated that patients with tubal factor infertility have a higher risk of OC [5]. Since the fallopian tubes are often affected by pelvic inflammatory disease (PID), it is therefore highly relevant to consider the potential role of infectious agents in OC. Previous reviews have focused on human papillomavirus (HPV) and OC, but this review is the first to include studies on all bacterial and viral infections as well as studies on pelvic inflammatory disease (PID).

## Material and methods

To review the existing literature on OC and infectious agents, we performed a systematic literature search by means of a database search for English language articles published in the period from 1980 to 2016. Using the Pubmed research tool, the keyword: “ovarian neoplasms” was by means of the Boolean logical “AND”, combined with the following keywords: “Chlamydia infections” (MeSH), “Neisseria gonorrhoeae” (MeSH), “Mycoplasma genitalium” (MeSH), “Papillomaviridae” (MeSH), “pelvic inflammatory disease” (MeSH). Correspondent searches were performed in EMBASE and Web of Science. The initial search identified 935 articles. After removal of 130 duplicates, 805 studies remained for further analysis. Contents were outside the scope of the present review in 740 articles that were excluded. The remaining 65 articles were subjected to full-text screening. Original studies, examining the association between infectious agents and ovarian cancer by use of tissue-based or serologic methods were included. Epidemiological studies examining the association between pelvic inflammatory disease and ovarian cancer were also included. Case-reports and reviews were excluded, leaving a total of 40 articles to be included in the review. More detailed information on the search strategy is given in the “Additional file 1” to this article.

## Viral agents investigated in relation to ovarian carcinogenesis

Table 1 summarises the viruses investigated in relation to OC, and their potential oncogenic mechanisms.

**Table 1:** Carcinogenic mechanisms of investigated viruses in relation to ovarian cancer

Virus	Viral family	Key viral transforming factors	Mechanisms	Associated cancers
Human papillomavirus	*Papillomaviridae*	E6 E7	Inhibition of apoptosis Immune evasion Cell cycle arrest Immune evasion [8, 10, 11]	Anal/rectal cancer Cervical cancer Oro-pharyngeal cancer (Ovarian cancer)^a^ Penile cancer Vaginal Vulvar cancer
Epstein bar virus	*Herpesviridae*	EBNA 1 EBNA 2 EBNA-3A Latent membrane protein 1	Inhibition of apoptosis B-cell growth transformation Cell cycle disruption Cell cycle disruption Inhibition of apoptosis Promotion of cell immortalisation [48, 49]	Burkitt lymphoma Gastric adenocarcinoma Hodgkin lymphoma Nasopharyngeal carcinoma Non-Hodgkin lymphoma (Ovarian cancer) Post-transplant lymphoproliferative disease
Cytomegalovirus	*Herpesviridae*	IE1/IE2	Inhibition of antiviral immune response Inhibition of apoptosis	Mucoepidermoid carcinoma of salivary glands (Ovarian cancer)
		US28 gene	Enhanced proliferative signaling [44, 45]	
John Cunningham virus/BK virus	*Polyomaviridae*	T-antigen/t-antigen	Cell cycle disruption Inhibition of apoptosis [56]	(Ovarian cancer)
Hepatitis C virus	*Flaviviridae*	(Indirect)	Chronic inflammation and oxidative stress Inhibition of antioxidant systems	Hepatocellular carcinoma (Ovarian cancer)
		(Direct) NS5B NOS2A NS3/4A protease	Cell cycle disruption Inhibition of apoptosis Inhibition of DDR response [51–53]	

*DDR* Double-stranded DNA Repair, *EBNA* Epstein Barr virus nuclear antigen, *IE* Immediate early genes, *NOS2A* Nitric oxide synthase 2A, *NS3/4A/5B* Nonstructural protein 3/4A/5B

^a^Causality is controversial and is still under investigation

### Human papillomavirus infection

HPV infection is a prerequisite for cervical cancer development and is implicated in a range of other cancer forms such as vulvar, penile, anal, and oropharyngeal cancers. To date, The International Agency for Research on Cancer has classified 12 HPV subtypes as human carcinogens: HPV16/18/31/33/35/39/45/51/52/56/58/59 [6]. In HPV-induced tumorigenesis, the viral oncogenes E6 and E7 are integrated into the host cell DNA, allowing viral oncoproteins to be expressed by the cell [7]. The carcinogenic mechanisms of these oncoproteins involve the ability of E6 to form a trimeric complex with P53 and the cellular E3 ubiquitin ligase, E6AP. This complex induces proteasomal degradation of P53, which leads to inhibition of cell-regulatory mechanisms and apoptosis and, therefore, permits continuous cell proliferation despite irreparable DNA damage [8]. E7 can cause degradation of retinoblastoma protein, which is an important regulator of the cell cycle [9]. Furthermore, E7 can interact with several cell cycle regulators including cyclins, cyclin-dependent kinases, CDKs-inhibitors, cullin 2, histone deacetylase, AP-1, E2F1, and E2F6 [10]. These actions enable the virus to resume the replication-competent state within the infected cells, which is essential for viral DNA synthesis. E6 and E7 can also exhibit immunomodulatory effects by downregulating expression of various inflammatory cytokines and by reducing the production of antiviral interferons [11].

HPV is the most studied infectious agent in relation to OC, and the available studies are summarised below.

Six case-control studies, with 11–50 cases of OC, were identified that examined the relation between HPV and OC [12–17]. The applied methods of analysis were either tissue-based methods or serologic assays and included in-situ hybridisation, immunohistochemistry, enzyme-linked immunosorbent serologic assay (ELISA), southern blot hybridisation technique, and polymerase chain reaction (PCR)-based technologies. Only one study found a statistically significant association between HPV and OC [12]. This study was the largest and included 50 cases with OC and a control group with 30 patients with non- malignant ovarian lesions. In-situ hybridisation and immunohistochemistry were used to detect the HPV 16 E6 gene in the tumour specimens. The highest detection rates were obtained by in-situ hybridisation, with a prevalence of 52% among cases as compared to 6.7% among controls (OR: 16.7 95% CI: 3.2–71.4). The remaining smaller studies, found no or non-significant associations [13–17].

Twenty-two *case-series* and *cross-sectional* studies reporting on the prevalence of HPV in OC tissue were identified (Table 2). HPV 16 was most frequently found and was reported in eight studies, giving a combined total of 60 cases. Ten studies did not find HPV in any of the samples [18–27]. The remaining studies reported a HPV prevalence ranging between 0.5 and 66.7% [28–39]. Interestingly, the reported HPV prevalence varied according to the geographical region. Among the 572 cases of OC in Europe and USA combined, 18 cases (3.1%) were positive for HPV DNA. Studies from the Middle East and Asia, with 347 combined cases of OC, reported 123 cases to be HPV DNA positive (35.4%).

**Table 2:** Studies reporting on HPV prevalence in ovarian cancer tissue

Author & publication year	Detection method	HPV genotypes found	Number of cases of EOC	Number of HPV positive cases	HPV prevalence (%)
Ingerslev et al. (2016) [28]	PCR	18	191	1	0.5
Al-Shabanah et al. (2013) [29]	PCR	16,18,45	100	42	42.0
Malisic et al. (2012) [30]	PCR	16	54	4	7.4
Bilyk et al. (2011) [31]	PCR	16,18	53	9	16.9
Idahl et al. (2010) [18]	PCR		52	0	0
Giordano et al. (2008) [32]	PCR	Not specified	50	1	2.0
Wentzensen et al. (2008) [19]	PCR		74	0	0
Atalay et al. (2007) [33]	PCR	16,33	94	8	8.5
Quirk et al. (2006) [20]	PCR		16	0	0
Yang et al. (2003) [34]	PCR	16,18	56	19	33.9
Ip et al. (2002) [35]	PCR	16,18	60	6	10
Li et al. (2002) [36]	PCR	Not specified	39	26	66.6
Antilla et al. (1999) [21]	PCR		98	0	0
Chen et al. (1999) [22]	PCR		20	0	0
Strickler et al. (1998) [37]	ELISA	16	16	1	6.2
Zimna et al. (1997) [38]	PCR	18	18	7	38.8
Anwar et al. (1996) [23]	PCR		3	0	0
Runnebaum et al. (1995) [24]	PCR		26	0	0
Trottier et al. (1995) [25]	PCR		22	0	0
Lai et al. (1994) [39]	PCR	16,18	18	11	61.1
Beckmann et al. (1991) [26]	PCR		18	0	0
de Villiers (1986) [27]	Southern blot hybridisation		7	0	0

Recently, sequencing data from The Cancer Genome Atlas (TCGA) database have been utilised to detect HPV oncogene expression in ovarian tumours. Using such data, Roos et al. concluded that six samples in 405 (1.5%) were positive for HPV18 oncogene expression [40]. However, Khoury et al. performed a similar analysis on 419 serous cystadenocarcinomas from the TCGA database and did not find signs of HPV infection [41]. Moreover, other studies present data that support potential HPV 18 contamination of the TCGA sequencing database and, consequently, question the validity of positive findings [42, 43].

### Cytomegalovirus infection

Cytomegalovirus (CMV) is under increasing focus as a carcinogenic virus. It is speculated that rather than initiating malignant transformation, CMV can enhance the growth and migration of tumour cells through a process called “oncomodulation” [44]. For instance, the formation of metastasis may be aided by the capacity of CMV to adhere to and disrupt the endothelial cell integrity by activating β1α5 integrin on the surface of tumour cells [45]. However, data also indicate that CMV has inherent antiapoptotic and cell cycle modulatory effects [45]. A single study has examined the relation between OC and CMV. The study detected CMV infection in 12 (50%) out of 24 cases with OC and in three (50%) out of six cases of borderline ovarian tumours. No signs of CMV infection were found in the control group consisting of normal ovarian tissue [13]. These findings have not been confirmed by others.

### Epstein-Barr virus and hepatitis C virus infection

Epstein-Barr virus (EBV) is present in more than 90% of the global adult population and causes latent infection in the host. [46] It exhibits tropism for epithelial cells, lymphocytes, and mesenchymal cells and is associated with a range of human cancers, including nasopharyngeal carcinoma (NPC) and lymphomas [47]. Its carcinogenic mechanisms include the actions of latent antigens that display effects similar to those of high-risk HPV oncogenes E6 and E7. An example is EBV nuclear antigen (EBNA)-1 that can induce proteasomal degradation of P53 and corresponding inhibition of apoptosis [48]. Likewise, EBNA-3A and -3C play an important role in cell cycle regulation and EBNA-2 antigen is suspected to cause primary B-cell growth transformation and maintain human B-cell immortalisation [49]. Hepatitis C virus (HCV) is another investigated microorganism in relation to OC. Chronic HCV infection greatly increases the risk of subsequent hepatocellular carcinoma [50]. The mechanisms are not fully understood but may entail different pathways. The chronic inflammation enhances a pro-carcinogenic microenvironment through the release of nitric oxide (NO) and reactive oxygen species (ROS). This process is further mediated by the ability of HCV to increase the cellular generation of NO and ROS [51] and by its capacity to weaken the antioxidant defence of the host. [52] A more direct pathway involves the ability of HCV to impair phosphorylation of the ataxia telangiectasia mutated (ATM) kinase substrate, histone 2A.X, which plays a role in initiating cellular response to double-stranded DNA breaks [53]. Despite the obvious carcinogenic potential of EBV and HCV, only a single study has investigated their potential role in relation to OC. However, this study did not detect EBV or HCV in 419 serous cystadenocarcinomas from the TCGA database [41].

### Polymyoma virus infection

Finally, a single study has analysed tissue samples for the presence of the BK virus and John Cunningham virus [18]. The oncogenic potential of polymyoma viruses has largely been attributed to viral proteins T- antigen and t-antigen through their interactions with various cellular proteins, including tumour suppressor proteins p53 and pRb [54, 55]. Furthermore, the capability of John Cunningham virus  T- antigen to transform rodent cells has previously been demonstrated [56]. In the included study, however, polymyoma viruses were not detected in any of the ovarian cancer tissue samples, nor in the benign controls.

## Bacterial agents investigated in relation to ovarian carcinogenesis

In relation to OC, previous research has focused on the potential association between *Chlamydia trachomatis (C. trachomatis), Mycoplasma genitalium (M. genitalium), and Neisseria gonorrhoeae (N. gonorrhoeae)* [13, 18, 57–61]. An overview of the potential carcinogenic mechanisms of these bacteria is provided in Table 3.

**Table 3:** Carcinogenic mechanisms of investigated bacteria in relation to ovarian cancer

Microorganism	Suspected mechanisms	Potentially associated cancers
*Chlamydia trachomatis*	Inhibition of apoptosis Production of reactive oxygen species Inhibition of DDR [62, 64]	Cervical squamous cell carcinoma Ovarian cancer
*Neisseria Gonorrhoeae*	DNA strand breaks. Inhibition of P53. Cell cycle disruption [68]	Bladder cancer Prostate cancer Ovarian cancer
*Mycoplasma Genitalium*	Acquisition of anchorage-independent growth Inhibition of apoptosis. Karyotypic entropy [69]	Ovarian cancer Prostate cancer

*DDR* Double-stranded DNA Repair

### Chlamydia trachomatis infection

*C. trachomatis* is a small, obligate intracellular bacterium that has developed several mechanisms to improve its own survival and replication within the host. It can upregulate the production of reactive oxygen species (ROS) through the Mitochondrial Nod-like Family Member NLRX1 [62]. ROS production is normally a cellular defence mechanism against invading microorganisms. However, *C. trachomatis* can subvert cellular defences and use ROS to improve its own growth through activation of caspase-1 [62]. The resulting increased levels of ROS can lead to double-stranded DNA breaks in the infected cell. Furthermore, *C. trachomatis* can impair the DNA damage response (DDR) through inhibition of DDR proteins pATM and 53BP1 [63]. Despite the DNA damage, the chlamydia-infected cells continue to proliferate due to increased MAPK signalling and cyclin E expression [63]. Finally, *C. trachomatis* can initiate proteasomal degradation of P53 through interaction with the phosphorylated ubiquitin ligase Murine Double Minute 2, leading to inhibition of apoptosis [64]. Combined, these mechanisms promote bacterial survival, but they can also potentially lead to genomic instability, disruption of the cell cycle, and inhibition of apoptosis, all of which are among the hallmarks of cancer [65]. Therefore, *C. trachomatis* is a suitable candidate for ovarian carcinogenesis. However, the literature on the subject is scarce, and an association between *C. trachomatis* and OC has been found in only two studies.

One case-control study was based on fresh ovarian tissues from 39 women undergoing laparotomy [13]. The tissue samples, which included 24 cases of OC, six borderline tumours, and nine normal ovaries, were tested for the presence of *C. trachomatis* DNA by PCR techniques. *C. trachomatis* DNA was detected in 87.5% of the malignant tumours and 50% of the borderline ovarian tumours as compared to none of the benign ovarian tissue samples (OR = 32 (95% CI: 3.33, 307.65).

A study by Ness et al. included 117 women with OC and 171 healthy controls [60]. Serologic testing for IgG antibodies of the extracellular elementary bodies (EB) of *C. trachomatis* was performed. Patients with EOC were more likely to have high levels of *C. trachomatis* EB than the controls in adjusted analysis. In a subgroup analysis, the probability of having OC was insignificantly increased in patients with the highest levels of IgG EB antibodies compared to the group with the lowest levels (OR = 1.9 95% CI 0.9–3.9). However, these findings are controversial, since four other serologic studies with a total of 626 cases of OC found no association [57–59, 61]. Likewise, a single tissue- based study that included 51 OC cases did not find an association [18].

### Neisseria gonorrhoeae & Mycoplasma genitalium infection

*N. gonorrhoeae* is a small, gram-negative diplococcus that infects epithelial cells and causes cervicitis/urethritis and PID. It has been associated with prostate and bladder cancer [66, 67]. One study has reported that *N. gonorrhoeae* can cause DNA strand breaks, affect the cell cycle progression, and can decrease the levels of P53 within infected cells [68].

*M. genitalium* is under increasing focus for its role in PID. Like *N. gonorrhoeae,* its propensity for malignant transformation of host cells is unclear. However, one study revealed that persistent infection of benign human prostate cells with *M. genitalium* resulted in increased migration/invasion [69]. Furthermore, it induced the acquisition of anchorage-independent growth, which allows cells to detach from the surrounding extracellular matrix and metastasise. Finally, it induced karyotypic entropy and malignant transformation proven by the formation of xenograft tumours in immune-compromised mice [69].

Very few studies have investigated the association between *M. genitalium* or *N. gonorrhoeae and OC.* Only one study reported an association, *M. genitalium* DNA being detected in 16 (59.3%) out of 27 samples by a combined PCR and ELISA technique. [70] However, in two separate studies, Idahl et al. found no association between *M. genitalium* or *N. gonorrhoeae* and OC [18, 57].

## Pelvic inflammatory disease

Microorganisms that cannot directly induce cellular transformation may still play a role in OC oncogenesis due to the paradoxical effect of the host inflammatory response. It has long been recognised that the cells involved in innate and adaptive immune responses are recruited to the site of tumorigenesis. Here, they can release a multitude of tumour promoting inflammatory cytokines, chemokines, and ROS that can potentially facilitate tumour cell migration, metastasis, and angiogenesis [65]. Cancer has aptly been described as “a wound that does not heal [71]”. Several conditions associated with pelvic inflammation such as perineal talc use and endometriosis have been demonstrated to increase the risk of OC [72]. Likewise, ovulation is speculated to induce focal damage and inflammation on the ovarian surface epithelium, and factors that reduce the number of lifetime ovulatory cycles have been shown to reduce the risk of OC [73]. Consequently, it has been speculated that PID could play a role in ovarian carcinogenesis, and the potential association between PID and OC have been investigated in seven case-control studies (Table 4). The majority of studies relied on patient-reported outcomes and identified cases of PID through interviews or questionnaires. Risch et al. reported a statistically significant association between PID and OC (odds ratio (OR) 1.53; 95% CI 1.10–2.13) [74]. Moreover, they found a dose-response effect in relation to repeated episodes of PID, implying a higher risk of OC with increasing episodes of PID (OR 1.88; 95% CI 1.13–3.12). A prospective, population-based study from Taiwan verified the diagnosis of PID by the International Statistical Classification of Diseases and Related Health Problems codes registered in a national database [75]. After 3 years of follow-up, a higher risk of OC was observed in the case group (hazard ratio (HR) 1.92; 95% CI 1.27–2.92)). In this study, the association was also stronger when analysis was restricted to cases exposed to more episodes of PID (HR 2.46; 95% CI 1.48–4.09)﻿﻿ [75]. However, a statistically significant association between PID and OC could not be confirmed in any of the remaining studies [72, 74, 76–79].

**Table 4:** Case-control studies on the association between PID and ovarian cancer

Author & publication year	Region	Data collection	Number of cases	Number of controls	Odds/Hazard- ratios (HR)
Rasmussen et al. (2013) [78]	Denmark	Interview	554	1.564	0.83 95% CI: 0.65–1.05
Lin et al. (2011) [75]	Taiwan	Database	67.936	135.872	1.92 95% CI: 1.27–2.92 (HR) 2.46 95% CI: 1.48–4.09^c^ (HR)
Merritt et al. (2007) [76]	Australia	Questionnaire	1.576	1.509	1.15 95% CI: 0.85–1.57
Ness et al. (2000) [72]	USA	Interview	616	1.367	1.3 95% CI: 0.6–2.5
Parazzini et al. (1996) [79]	Italy	Questionnaire/interview	971	2.758	0.7 95% CI: 0.4–1.3
Risch et al. (1995) [74]	Canada	Interview	450	564	1.53 95% CI: 1.10–2.13^a^ 1.88 95% CI: 1.13–3.12^b^
Shu et al. (1989) [77]	Hong Kong	Interview	172	172	3.0 95% CI: 0.3–30.2

^a^One episode of PID

^b^Two or more episodes of PID

^c^Five or more episodes of PID

## Discussion

The reviewed studies were very heterogeneous in terms of study population, study design, and the analysis methods used.

However, based on the reviewed studies, it is fair to conclude that high-risk HPV is unlikely to be associated with OC in Western countries. Interestingly, a higher prevalence was consistently reported in Asia and the Middle East. This finding is supported by previous reviews focusing on HPV and ovarian cancer [80, 81] and a potential association may exist in these regions. The geographic variation could be caused by varying potency of HPV strains. Thus, it has been demonstrated that latent infection of the cervix uteri with the non- European variant of HPV 16 is associated with a 2- to 9-fold higher risk of cancer or high- grade cancer precursors, compared to infection with the European variant [82]. Differing genetic predispositions between ethnical groups may also be a factor because polymorphisms of the TP 53 gene or the promoter of the tumour necrosis factor alpha gene have been associated with increased vulnerability to HPV oncogenes [83, 84]. Lastly, environmental or life-style factors could play a role. For instance, the global incidence of EBV infection among adults is estimated to be over 90%, and there is strong evidence linking EBV infection to the development of NPC [47]. Despite the ubiquity of infection, the incidence of NPC is very low in most regions. However, dramatically elevated rates are observed in certain parts of Asia, and dietary factors, such as the intake of salt-preserved fish, are thought to play a role [85]. This underlines that, even though infection with oncogenic viruses is common, cancer is a rare event that can arise from a combination of genetic, environmental, and lifestyle factors.

Most of the epidemiological studies found an association between OC and PID, but only two studies reported a statistically significant association [74, 75]. It is noteworthy, however, that these studies also identified a dose- response effect. This is one of the key points in Hill’s criteria for causation that state that a biological gradient must be present, with more exposure to the suspected agent leading to a larger effect [86]. These findings are supported by studies that found evaluated markers of inflammation such as CRP and interleukins to be significantly associated with an increased risk of OC [87]. A possible limitation to the reviewed studies is that they relied primarily on patient-reported outcomes, which may be subject to recall bias. Furthermore, due to the potential subclinical course of PID, patients can be unaware of previous episodes. This is supported by studies that have demonstrated a relatively high seroprevalence of chlamydia antibody titres in women that reported no history of PID, and this issue would tend to produce bias towards the null [88, 89].

Interestingly, CMV was reported in 50% of cases in a single study, but this finding requires confirmation in future and larger studies. No other viral agent was detected.

The only detected bacteria were *C. trachomatis and M. genitalium.* The results of this review do not support an association between *C. trachomatis* and OC, the majority of studies reporting negative results*.* The association between *M. genitalium* and OC was investigated and found in only a single study and more research is needed.

Several factors may account for the conflicting results. First of all, there may be no connection between the investigated infectious agents and OC. Secondly, the heterogeneity of analysis methods, study populations, and study designs may play a part. However, other explanations must be discussed. Importantly, it is unclear to what extent signs of microbiological agents are present in a tumour that arises decades after infection. For some oncogenic viruses, like high-risk HPV, expression of viral oncogenes is obligate for the maintenance of the malignant phenotype [90]. In these cases, viral genes will remain integrated and detectable in tumour cell DNA. But another potential mechanism is debated in the “hit and run” hypothesis. Here, integration of viral transforming genes and potential epigenetic reprogramming of host cells initiate tumorigenesis, but the viral gene expression is subsequently lost during neoplastic development [91]. Accordingly, it has been demonstrated that CMV oncogenes IE1 and IE2 can cooperate with adenovirus E1A gene to transform rat kidney cells. However, expression of the transforming oncogenes was absent in the clonal cell lines from the transformed foci [92].

Finally, other oncogenic agents act more indirectly by inducing chronic inflammation that may function as an initiator or promoter of carcinogenesis [93]. Consequently, the involved pathogen may no longer be present when cancer is diagnosed. For instance, despite the established role of HCV in hepatocellular carcinoma, only a minority of transformed hepatocytes contain viral RNA [94].

These examples highlight the fact that the abscense of microbiological agents in tumour tissue does not rule out their potential role in the transformation of host cells. These issues seem to strengthen the need for serologic studies. However, serologic methods are limited by the fact that antibody levels are likely to decline over time. In a study from Finland, 43% of women had declining *C. trachomatis* antibody titres 6 years after the diagnosis of PID [95]. Moreover, not all patients with prior chlamydial infection will have detectable antibodies [96].

These obstacles call for a revised strategy in the attempt to uncover the potential role of infectious agents in OC. It is fair to assume that signs of microbiological presence will be easier to detect when the interval between primary infection and investigation is short. Therefore, it seems more relevant to investigate precursor lesions for the presence of potential microbiological agents [91]. Until recently, this has been impossible due to the lack of a clearly defined premalignant lesion to OC. However, this has changed because increasing evidence indicates that STIC lesions in the distal fallopian tubes are precursor lesions to serous epithelial OC, which constitutes the majority of OC cases [3]. Yet, no explanation has been formulated as to why STIC lesions arise. Since the fallopian tubes are often affected and damaged by chronic PID, it is biological plausible that pathogens with transforming capacities, or the chronic inflammation they induce, could lead to subsequent neoplastic transformation. We therefore recommend that future studies focus on the detection of bacterial or viral agents in fallopian tube tissue samples with STIC lesions verified through the “Sectioning and Extensively Examining of the Fimbriated end” protocol.

## Conclusion

The reviewed articles indicate a potential association between PID and OC. An association between OC and high-risk HPV genotypes may exist in Asia, whereas an association in Western countries seems unlikely due to the low reported prevalence. Two studies reported an association between bacterial agents and OC, but the majority of bacterial studies reported negative findings. However, the available literature was scarce and more studies are warranted.

## Additional file

Additional file 1: Systematic literature search. (DOCX 37 kb)

## Abbreviations

ATM: Ataxia telangiectasia mutated
*C. trachomatis*: *Chlamydia trachomatis*
CI: Confidence interval
CMV: Cytomegalovirus
DDR: Double-stranded DNA Repair.
EBNA: Epstein bar virus nuclear antigen
EBV: Epstein bar virus
ELISA: Enzyme-linked immunosorbent serologic assay
HCV: Hepatitis C virus
HPV: Human papillomavirus
HR: Hazard ratio
IE: Immediate early genes
*M. genitalium*: *Mycoplasma genitalium*
*N. Gonorrhoeae*: *Neisseria gonorrhoeae*
NO: Nitric oxide
NOS2A: Nitric oxide synthase 2A
NPC: Nasopharyngeal carcinoma
NS3/4A: Nonstructural protein 3/4A
OC: Ovarian cancer
PCR: Polymerase chain reaction
PID: Pelvic inflammatory disease
ROS: Reactive oxygen species
STIC: Serous tubal intraepithelial carcinoma
TCGA: Cancer Genome Atlas database
