# Supplementary material for: The potential role of infectious agents and pelvic inflammatory disease in ovarian carcinogenesis
Source: Infect Agent Cancer. 2017 May 18;12:25. doi: 10.1186/s13027-017-0134-9 (PMC5437405; doi:10.1186/s13027-017-0134-9)
Supplement: Additional file 1: — Systematic literature search. (DOCX 37 kb) [file 13027_2017_134_MOESM1_ESM.docx]

**Systematic literature search**

On September 9th 2016, the databases MEDLINE, EMBASE, and Web of Science were searched for English language articles published in the period of 1980-2016. The initial search strategy was developed in collaboration with a research librarian at Aarhus University, Denmark. Using the Pubmed research tool, the keyword: “ovarian neoplasms” was by means of the Boolean logical “AND”, combined with the following keywords: “Chlamydia infections” (MeSH), “Neisseria gonorrhoeae” (MeSH), “Mycoplasma genitalium” (MeSH), “Papillomaviridae” (MeSH), “pelvic inflammatory disease” (MeSH). Initially, no constraints regarding article types were employed. Correspondent searches were performed in EMBASE and Web of Science. A free- text search was also performed to identify non-indexed articles. All retrieved articles were imported into Refworks (RefWorks-COS, ProQuest, UK http://www.refworks.com/). Exact and close matching duplicates were identified by a Refworks algorithm and were removed after individual assessment by the reviewers. Bibliographies were systematically studied to identify additional papers. Original studies, examining the association between infectious agents and ovarian cancer by use of tissue-based or serologic methods were included. Epidemiological studies examining the association between pelvic inflammatory disease and ovarian cancer were also included. Case-reports and reviews were excluded.

The literature search identified 931 articles. Four additional articles were identified by systematically studying bibliographies[1-4]. After removal of 130 duplicates, 805 studies remained for further analysis. Contents were outside the scope of the present review in 740 articles that were excluded. The remaining 65 articles were subjected to full-text screening. Fourteen, non- original, articles were excluded, including nine reviews, two case-reports and three commentary articles. Six additional articles were outside the scope of the review and were excluded. One article was excluded since the same data was included in two studies[5]. We suspected that this was also the case for two other studies[6,7]. Since we were unable to clarify this we decided to include the study that was published first[6]. Three additional articles were excluded: one due to lack of differentiation between endometrial and ovarian cancer[8], one that only included patients with low malignant potential tumors[9] and finally one was excluded since it was later retracted by the authors due to a possible contamination of the samples[10]. The remaining 40 articles met the inclusion criteria for this review[1-4,6,11-45] (see figure 1).

Figure S1. Database search

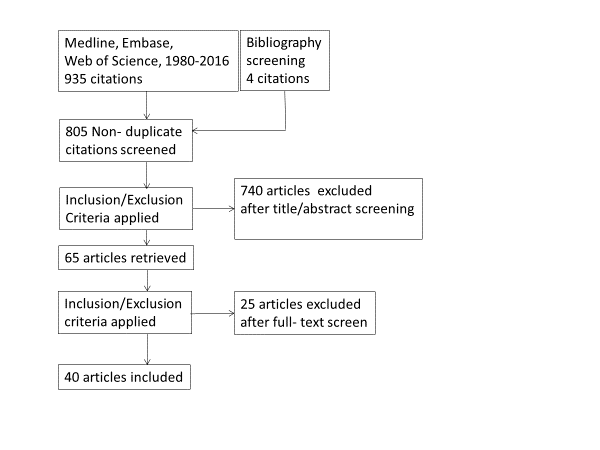


(1) Shu XO, Brinton LA, Gao YT, et al. Population-based case-control study of ovarian cancer in Shanghai. Cancer Res 1989; 49: 3670-4.

(2) de Villiers E, Schneider A, Gross G, et al. Analysis of benign and malignant urogenital tumors for human papillomavirus infection by labelling cellular DNA. Med Microbiol Immunol (Berl ) 1986; 174: 281-6.

(3) Anwar K, Nakakuki K, Imai H, et al. Infection of human papillomavirus (HPV) and p53 over-expression in human female genital tract carcinoma. JOURNAL-PAKISTAN MEDICAL ASSOCIATION 1996; 46: 220-4.

(4) Ness RB, Grisso JA, Cottreau C, et al. Factors related to inflammation of the ovarian epithelium and risk of ovarian cancer. Epidemiology 2000; 11: 111-7.

(5) Malisic E, Jankovic R, Jakovljevic K, et al. TP53 gene status and human papilloma virus infection in response to platinum plus taxane-based chemotherapy of epithelial ovarian carcinomas. J BUON 2011; 16: 701-7.

(6) Al-Shabanah OA, Hafez MM, Hassan ZK, et al. Human papillomavirus genotyping and integration in ovarian cancer Saudi patients. Virol J 2013; 10: 343,422X-10-343.

(7) Al-Shabanah OA, Hafez MM, Hassan ZK, et al. Methylation of SFRPs and APC genes in ovarian cancer infected with high risk human papillomavirus. Asian Pac J Cancer Prev 2014; 15: 2719-25.

(8) Konidaris S, Kouskouni EE, Panoskaltsis T, et al. Human papillomavirus infection in malignant and benign gynaecological conditions: a study in Greek women. Health Care Women Int 2007; 28: 182-91.

(9) McLellan R, Buscema J, Guerrero E, et al. Investigation of ovarian neoplasia of low malignant potential for human papillomavirus. Gynecol Oncol 1990; 38: 383-5.

(10) Kaufman RH, Adam E, Adler-Storthz K. Absence of human papillomavirus (HPV) DNA sequences in epithelial ovarian carcinoma. Gynecol Oncol 1990; 37: 148.

(11) Shanmughapriya S, Senthilkumar G, Vinodhini K, et al. Viral and bacterial aetiologies of epithelial ovarian cancer. Eur J Clin Microbiol Infect Dis 2012; 31: 2311-7.

(12) Idahl A, Lundin E, Jurstrand M, et al. Chlamydia trachomatis and Mycoplasma genitalium plasma antibodies in relation to epithelial ovarian tumors. Infect Dis Obstet Gynecol 2011; 2011: 824627.

(13) Idahl A, Lundin E, Elgh F, et al. Chlamydia trachomatis, Mycoplasma genitalium, Neisseria gonorrhoeae, human papillomavirus, and polyomavirus are not detectable in human tissue with epithelial ovarian cancer, borderline tumor, or benign conditions. Am J Obstet Gynecol 2010; 202: 71.e1,71.e6.

(14) Ness RB, Shen C, Bass D, et al. Chlamydia trachomatis serology in women with and without ovarian cancer. Infect Dis Obstet Gynecol 2008; 2008: 219672.

(15) Wong A, Maclean AB, Furrows SJ, et al. Could epithelial ovarian cancer be associated with chlamydial infection? Eur J Gynaecol Oncol 2007; 28: 117-20.

(16) Ness R.B. Ovarian cancer, inflammation and endometriosis. CME J Gynecol Oncol 2003; 8: 33-40.

(17) Martin DC, Khare VK, Miller BE, et al. Association of positive Chlamydia trachomatis and Chlamydia pneumoniae immunoglobulin-gamma titers with increasing age. J Am Assoc Gynecol Laparosc 1997; 4: 583-6.

(18) Kuscu E, Ozdemir BH, Erkanli S, et al. HPV and p53 expression in epithelial ovarian carcinoma. Eur J Gynaecol Oncol 2005; 26: 642-5.

(19) Wu QJ, Guo M, Lu ZM, et al. Detection of human papillomavirus-16 in ovarian malignancy. Br J Cancer 2003; 89: 672-5.

(20) Lai CH, Hsueh S, Lin CY, et al. Human papillomavirus in benign and malignant ovarian and endometrial tissues. Int J Gynecol Pathol 1992; 11: 210-5.

(21) Leake JF, Woodruff JD, Searle C, et al. Human papillomavirus and epithelial ovarian neoplasia. Gynecol Oncol 1989; 34: 268-73.

(22) Hisada M, van den Berg BJ, Strickler HD, et al. Prospective study of antibody to human papilloma virus type 16 and risk of cervical, endometrial, and ovarian cancers (United States). Cancer Causes Control 2001; 12: 335-41.

(23) Ingerslev K, Hogdall E, Skovrider-Ruminski W, et al. High-risk HPV is not associated with epithelial ovarian cancer in a Caucasian population. Infectious Agents and Cancer 2016; 11: 1.

(24) Malisic E, Jankovic R, Jakovljevic K. Detection and genotyping of human papillomaviruses and their role in the development of ovarian carcinomas. Arch Gynecol Obstet 2012; 286: 723-8.

(25) Bilyk OO, Pande NT, Buchynska LG. Analysis of p53, p16(INK4a), pRb and Cyclin D1 expression and human papillomavirus in primary ovarian serous carcinomas. Exp Oncol 2011; 33: 150-6.

(26) Giordano G, D'Adda T, Gnetti L, et al. Role of human papillomavirus in the development of epithelial ovarian neoplasms in Italian women. J Obstet Gynaecol Res 2008; 34: 210-7.

(27) Wentzensen N, du Bois A, Kommoss S, et al. No metastatic cervical adenocarcinomas in a series of p16INK4a-positive mucinous or endometrioid advanced ovarian carcinomas: an analysis of the AGO Ovarian Cancer Study Group. Int J Gynecol Pathol 2008; 27: 18-23.

(28) Atalay F, Taskiran C, Taner MZ, et al. Detection of human papillomavirus DNA and genotyping in patients with epithelial ovarian carcinoma. J Obstet Gynaecol Res 2007; 33: 823-8.

(29) Quirk JT, Kupinski JM, DiCioccio RA. Analysis of ovarian tumors for the presence of human papillomavirus DNA. J Obstet Gynaecol Res 2006; 32: 202-5.

(30) Yang HJ, Liu VW, Tsang PC, et al. Comparison of human papillomavirus DNA levels in gynecological cancers: implication for cancer development. Tumour Biol 2003; 24: 310-6.

(31) Ip SM, Wong LC, Xu CM, et al. Detection of human papillomavirus DNA in malignant lesions from Chinese women with carcinomas of the upper genital tract. Gynecol Oncol 2002; 87: 104-11.

(32) Li T., Lu Z.-M., Guo M., et al. p53 Codon 72 polymorphism (C/G) and the risk of human papillomavirus-associated carcinomas in China. Cancer 2002; 95: 2571-6.

(33) Anttila M, Syrjanen S, Ji H, et al. Failure to demonstrate human papillomavirus DNA in epithelial ovarian cancer by general primer PCR. Gynecol Oncol 1999; 72: 337-41.

(34) Chen TR, Chan PJ, Seraj IM, et al. Absence of human papillomavirus E6-E7 transforming genes from HPV 16 and 18 in malignant ovarian carcinoma. Gynecol Oncol 1999; 72: 180-2.

(35) Strickler H.D., Schiffman M.H., Shah K.V., et al. A survey of human papillomavirus 16 antibodies in patients with epithelial cancers. Eur J Cancer Prev 1998; 7: 305-13.

(36) Zimna K, Poreba E, Kedzia W, et al. Human papillomavirus (HPV) in upper genital tract carcinomas of women. Eur J Gynaecol Oncol 1997; 18: 415-7.

(37) Runnebaum IB, Maier S, Tong XW, et al. Human papillomavirus integration is not associated with advanced epithelial ovarian cancer in German patients. Cancer Epidemiol Biomarkers Prev 1995; 4: 573-5.

(38) Trottier AM, Provencher D, Mes-Masson AM, et al. Absence of human papillomavirus sequences in ovarian pathologies. J Clin Microbiol 1995; 33: 1011-3.

(39) Lai CH, Wang CY, Lin CY, et al. Detection of human papillomavirus RNA in ovarian and endometrial carcinomas by reverse transcription/polymerase chain reaction. Gynecol Obstet Invest 1994; 38: 276-80.

(40) Beckmann AM, Sherman KJ, Saran L, et al. Genital-type human papillomavirus infection is not associated with surface epithelial ovarian carcinoma. Gynecol Oncol 1991; 43: 247-51.

(41) Rasmussen CB, Faber MT, Jensen A, et al. Pelvic inflammatory disease and risk of invasive ovarian cancer and ovarian borderline tumors. Cancer Causes Control 2013; 24: 1459-64.

(42) Lin HW, Tu YY, Lin SY, et al. Risk of ovarian cancer in women with pelvic inflammatory disease: a population-based study. Lancet Oncol 2011; 12: 900-4.

(43) Merritt MA, Green AC, Nagle CM, et al. Talcum powder, chronic pelvic inflammation and NSAIDs in relation to risk of epithelial ovarian cancer. Int J Cancer 2008; 122: 170-6.

(44) Parazzini F, La Vecchia C, Negri E, et al. Pelvic inflammatory disease and risk of ovarian cancer. Cancer Epidemiol Biomarkers Prev 1996; 5: 667-9.

(45) Risch HA, Howe GR. Pelvic inflammatory disease and the risk of epithelial ovarian cancer. Cancer Epidemiol Biomarkers Prev 1995; 4: 447-51.
